# Gap Junctions in the Bone Marrow Lympho-Hematopoietic Stem Cell Niche, Leukemia Progression, and Chemoresistance

**DOI:** 10.3390/ijms21030796

**Published:** 2020-01-25

**Authors:** Abhishek K. Singh, Jose A. Cancelas

**Affiliations:** 1 Division of Experimental Hematology and Cancer Biology, Cincinnati Children’s Hospital Medical Center, 3333 Burnet Ave., Cincinnati, OH 45229, USA; abhishek.singh@uc.edu; 2 Hoxworth Blood Center, University of Cincinnati Academic Health Center, 3333 Burnet Ave., Cincinnati, OH 45229, USA

**Keywords:** gap junction, connexin, hematopoietic stem cells and progenitors, stromal cells, niche, leukemia, mitochondria, reactive oxygen species, tunneling nanotubes

## Abstract

The crosstalk between hematopoietic stem cells (HSC) and bone marrow (BM) microenvironment is critical for homeostasis and hematopoietic regeneration in response to blood formation emergencies after injury, and has been associated with leukemia transformation and progression. Intercellular signals by the BM stromal cells in the form of cell-bound or secreted factors, or by physical interaction, regulate HSC localization, maintenance, and differentiation within increasingly defined BM HSC niches. Gap junctions (GJ) are comprised of arrays of membrane embedded channels formed by connexin proteins, and control crucial signaling functions, including the transfer of ions, small metabolites, and organelles to adjacent cells which affect intracellular mechanisms of signaling and autophagy. This review will discuss the role of GJ in both normal and leukemic hematopoiesis, and highlight some of the most novel approaches that may improve the efficacy of cytotoxic drugs. Connexin GJ channels exert both cell-intrinsic and cell-extrinsic effects on HSC and BM stromal cells, involved in regenerative hematopoiesis after myelosuppression, and represent an alternative system of cell communication through a combination of electrical and metabolic coupling as well as organelle transfer in the HSC niche. GJ intercellular communication (GJIC) in the HSC niche improves cellular bioenergetics, and rejuvenates damaged recipient cells. Unfortunately, they can also support leukemia proliferation and survival by creating leukemic niches that provide GJIC dependent energy sources and facilitate chemoresistance and relapse. The emergence of new strategies to disrupt self-reinforcing malignant niches and intercellular organelle exchange in leukemic niches, while at the same time conserving normal hematopoietic GJIC function, could synergize the effect of chemotherapy drugs in eradicating minimal residual disease. An improved understanding of the molecular basis of connexin regulation in normal and leukemic hematopoiesis is warranted for the re-establishment of normal hematopoiesis after chemotherapy.

## 1. Introduction

**Figure 1:**
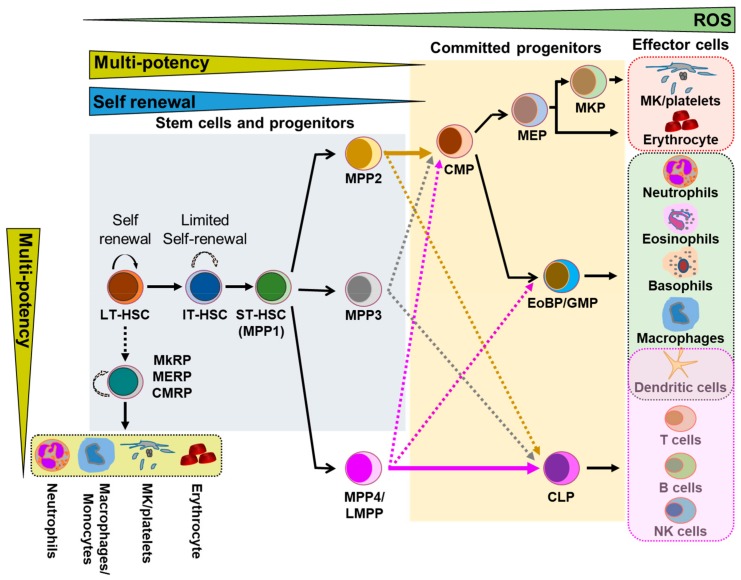
Hematopoietic stem cells hierarchy. The hematopoietic stem cells (HSC) pool is highly heterogeneous, containing long-term hematopoietic stem cell (LT-HSC), intermediate-term hematopoietic stem cells (IT-HSC), and short-term hematopoietic stem cells (ST-HSC/MPP1). These cells are multipotent with differing self-renewal abilities. HSC differentiate into MPP2, MPP3, and MPP4/LMPP subpopulations. MPP2 and MPP3 cells are myeloid biased, and give rise to common myeloid progenitors (CMP), which can further differentiate into mature hematopoietic cells via megakaryocyte-erythrocyte progenitors (MEP) and granulocyte-macrophage progenitors (GMP) stages. MPP4 primarily differentiate into the common lymphoid progenitor (CLP), followed by mature T, B, and NK cells. In the myeloid bypass model, loss of HSC self-renewal generates myeloid-restricted repopulating progenitors, which can be megakaryocyte repopulating progenitors (MkRP), megakaryocyte-erythrocyte repopulating progenitors (MERP), and common myeloid repopulating progenitors (CMRP), and give rise to erythrocytes, platelets, neutrophils, and monocytes. MPP-Multi potent progenitors, LMPP-lymphoid-primed multipotent progenitors, EoBP—eosinophil basophil progenitors, MKP—megakaryocyte progenitors, MK-megakaryocytes.

Lifelong production of blood cells and the robust regenerative capacity of lympho-hematopoiesis depend on hematopoietic stem cell (HSC) self-renewal, proliferation, and differentiation. HSC reside in a highly specialized bone marrow (BM) microenvironment (BMME), also called “niche”, that helps in maintaining HSC quiescence and long-term repopulating activity. In steady-state or stress-adapted hematopoiesis, long-term HSC (LT-HSC), capable of long-term self-renewal and multipotential differentiation ability, can differentiate into short-term HSC (ST-HSC) followed by multi-potent progenitors (MPP), which generate a series of uni- or oligo-potent lineage-committed progenitors, and give rise to all mature blood cells [1,2,3] (Figure 1). The fate of HSC is tightly regulated by a combination of cell-intrinsic (transcriptional and epigenetic regulators) and cell-extrinsic factors (soluble growth factors, cytokines, microbial ligands, and adhesive interactions) [4,5]. Several studies have demonstrated cell-to-cell interactions between HSC and the surrounding niche cells (endothelial cells, stromal cells, and osteoblasts), which are essential for HSC localization, maintenance, and differentiation [6,7,8,9]. Gap junctions (GJ) are complexes of intercellular channels formed between the juxtaposed membranes of two adjacent cells which allow the intercellular transfer of ions, metabolites, soluble factors, and secondary messenger molecules smaller than 1200 Da [10,11,12,13]. A growing body of work has detailed the importance of GJ mediated intercellular communication (GJIC) in the regulation of signaling pathways required for HSC survival, proliferation, and fate decisions [8,14,15,16,17,18].

Niche environment regulates both normal and malignant hematopoiesis by offering needed nutrients. Leukemia cells, however, modify their surrounding niche into an abnormal but favorable environment, which outcompetes with the native niches for HSC cell localization, and fails to preserve the normal HSC pool size by impeding the differentiation at the HSC-progenitor transition [19,20]. A large cohort of experimental studies suggest that cancer cells consume high levels of glucose and largely depend on aerobic glycolysis to generate adenosine triphosphate (ATP), however, leukemic cells display a metabolic shift and are primarily dependent on mitochondrial oxidative phosphorylation for survival [21,22]. The metabolic reprogramming in leukemia cells is influenced by crosstalk with surrounding BMME, and cumulative evidence suggests that the BM stromal cells (BMSC) either indirectly through secreted factors, or directly by cell-to-cell interactions via GJ-mediated channels, regulate leukemia initiation, progression, and relapse [23,24]. This review intends to discuss the role of GJ in both normal and leukemic hematopoiesis, and highlight some of the most novel approaches that may improve the efficacy of cytotoxic drugs. We also highlight possible directions for the development of successful tumor targeting strategies. 

## 2. Gap Junctions, Connexons, and Connexins

**Figure 2:**
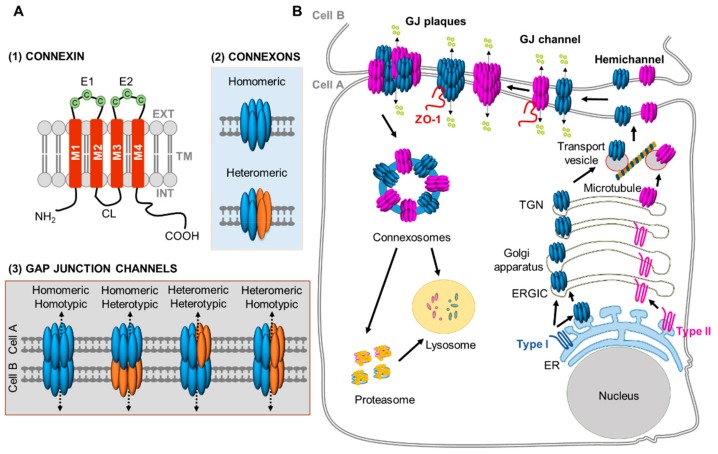
Cell biology and structure of connexins. (**A**) Structural organization of a connexin, a connexon, and a gap junction channel. (1) Connexins contain four transmembrane domains (M1, M2, M3, and M4), two extracellular loops (E1 and E2), and three intercellular domains i.e., amino-terminal (NT), the cytoplasmic loop (CL), and the carboxy-terminal (CT) domains. (2) Connexins form hexamers called hemichannel/connexon. Connexons formed by identical connexin isoforms form homomeric connexons, whereas connexons composed of two or more different types of connexin isoforms form heteromeric connexons. (3) Connexons form functional gap junction (GJ) channels by interacting with either identical homomeric or heteromeric connexons of adjacent cells, forming homotypic GJ channels, or with different homomeric or heteromeric connexons, forming heterotypic GJ channels. C-Cysteine, EXT—Extracellular, INT—Intracellular, TM—Transmembrane. (**B**) Schematic representation of the steps of connexin gap junction channel synthesis, assembly, and degradation. Biosynthesis of connexin polypeptides starts at endoplasmic reticulum (ER) membranes, and depending on the type of connexin (Type 1 or Type 2), hexameric oligomerization occurs either in the ER-Golgi intermediate compartment (ERGIC) or the trans-Golgi network (TGN). Hemichannels are subsequently transported to the plasma membrane along microtubules, where they pair with the hemichannels of adjacent cells to form a complete intercellular GJ channels. Individual GJ channels at the plasma membrane aggregate to form the GJ plaques, which may contain homomeric and heteromeric GJ channels. Connexin binding proteins zonula occludens-1 (ZO-1) play a role in regulating GJ assembly and plaque size. GJ or fragments of GJ are internalized as unique double-membrane connexosomes, and degraded by lysosomes, ubiquitin-dependent proteasomes, or both.

GJ consist of aggregates of transmembrane hemichannels or connexons that adjoin head-to-head with the connexon of adjacent cells. Each hemichannel consists of six connexins (Cx), which contain four transmembrane domain (M1, M2, M3, and M4), two extracellular loops (E1 and E2), and three intercellular domains, including the amino-terminal (NT), the cytoplasmic loop (CL), and the carboxy-terminal (CT) domains [10,11,12,13] (Figure 2A). Connexins are highly conserved chordate proteins of about 200–500 amino acids, and 21 different connexins in humans and 20 different connexins in mice have been identified [10,11,12,13]. Additionally, based on the similarity of amino acid sequences, connexins are subdivided into five different subfamilies (α, β, γ, δ, and ɛ) [10]. Most mammalian cells express connexins, and there exists strong evidence that many cells simultaneously express more than one connexin [25,26,27]. Although many connexins are expressed at the mRNA level, the protein expression may not reach functional levels in specific types of cells. This is especially important in BM hematopoiesis where the state of activation and/or differentiation of cell subtypes is highly dependent on the level of expression and/or function of connexins [15,17,28,29,30,31,32]. 

Docking of the hemichannels between two neighboring cells is a quick and highly regulated process, and essential for the formation of a complete GJ channel, providing an intercellular route for the passage of small ions and metabolites between communicating cells. Hemichannels which are composed of identical Cx isoforms are known as homomeric connexons, and hemichannels containing different types of Cx isoforms are referred to as heteromeric connexons. The association of two identical homomeric or heteromeric connexons forms a homotypic GJ, while the docking of two different homomeric hexamers, or a homomeric and a heteromeric hexamer, or two different heteromeric hexamers form heterotypic GJ channels (Figure 2A).

### Connexin Biosynthesis and Turnover

GJ intercellular communication (GJIC) is regulated by connexin biosynthesis, transport, and assembly, as well as their internalization and degradation. The half-life of connexins is relatively short, ranging from 1.5 to 5 h [33], however, certain connexins may survive longer e.g., Cx46, which may survive throughout the lifetime of expressing cells, or Cx30, which survives during the process of differentiation of some non-hematopoietic cell types [34,35]. Connexins are formed on the membrane of the endoplasmic reticulum (ER), where they undergo hexameric oligomerization and intramolecular disulfide bond formation, critical for the docking and functional assembly of the GJ channel [36,37,38]. However, substantial evidence suggests that the oligomerization of Cx43, and possibly Cx46, does not occur within the ER but mainly occurs in the trans-Golgi network (TGN) [39] (Figure 2B). The post-translational processing of Cx43 and Cx46 might be associated with the ER quality-control pathways and prevent the premature opening of connexons in the ER membrane. The completely processed connexin hemichannels finally move to the cell membrane through interaction with microtubules [40,41]. Many GJIC cluster to form a tightly packed semi-crystalline arrays referred as GJ plaques. The outer edges of the GJ plaque are composed of newly synthesized connexons, while the older GJ channels are found in the center of the plaque, where they get internalized and destined for degradation [42,43,44]. The degradation of GJ plaques occurs via the formation of connexosomes (annular junction), where an entire GJ or a fragment of GJ are removed by lysosomal, phago-lysosomal, autophagy, or ubiquitin dependent proteasomal pathways [33,45]. Post-translational modifications, such as phosphorylation, may trigger the internalization and proteasomal degradation of GJ in different cells. In addition, the tight junction-associated protein, zonula occludens-1 (ZO-1) binds connexins, including Cx43 and Cx45, via a PDZ domain and regulates GJ assembly and the size of plaques [46,47,48] (Figure 2B). While we have advanced our understanding of the life-cycle of connexins and GJ, there is consensus that certain processes are especially relevant in specific cell types where the subcellular localization of connexins and GJIC may result in function specialization and specific secondary messenger gating, cell adhesion, scaffolding, or activation of context-dependent signalomes, that are relevant in health and disease.

## 3. Involvement of GJIC in Hematopoiesis 

In the 1980s and 1990s, a number of reports identified the presence of GJ in BM and in in vitro hematopoietic model systems of cell-to-cell interaction [49,50,51,52,53]. The existence of GJ in hematopoietic cells collected directly from mouse BM was first demonstrated by Campbell, who used a tannic acid and glutaraldehyde fixative and electronic microscopy techniques to identify GJ between macrophages and stem cells, monocytes, erythroblasts, and neutrophilic precursors, including promyelocytes, myelocytes, and metamyelocytes [54]. Yamazaki et al. provided elegant ultrastructural studies on the microanatomical location of GJ and the effect of hematopoietic Kit mutations on the upregulation of GJ [55,56,57,58,59]. Rosendaal and colleagues further described anatomical and functional details of the expression and role of GJ and Cx43 in lympho-hematopoietic tissues [18,60,61,62,63,64,65,66,67,68,69] and Evans and colleagues identified the expression and function of connexins in lymphocytes [70,71,72,73,74,75]. The existence of functional GJ channels in BMSC, and in between hematopoietic stem and progenitor cells (HSPC) and BMSC in both normal and stress hematopoiesis [18,63,68], strongly supported a role for GJIC in hematopoietic homeostasis and regeneration, but as of 2010, there was no clear mechanistic understanding of how connexins control HSC activity in the BM.

### 3.1. Connexins in HSC 

The expression of GJ protein in a hematopoietic compartment is indispensable for both steady-state and stress hematopoiesis [15,28,29]. Transcript analysis has identified the presence of Cx43, Cx45, Cx31, Cx31.1, Cx32, Cx37, and Cx50 in the primitive stem cell compartment, primarily in LT-HSC [15,28,29], while their expression was downregulated during differentiation to progenitors [28]. In normal hematopoiesis, Cx32 is expressed solely in the primitive HSC compartment (LSK and LK cells), where it maintains HSC quiescence and stemness [29], and protects them from chemical abrasion and leukemogenic impacts [76]. Deficiency of Cx32 is associated with decreased leukocytes and platelets in peripheral blood, with increased cell proliferation and content of hematopoietic progenitors in the BM [29,76] (Table 1). By contrast, inhibition of Cx32 by mimetic peptides did not phenocopy the HSPC proliferation seen in Cx32 deficient mice. This could be because Cx32 regulates hematopoietic progenitor proliferation through cell-intrinsic intracellular signaling, and is independent of functional Cx32 GJ channel or hemichannels. 

**Figure 3:**
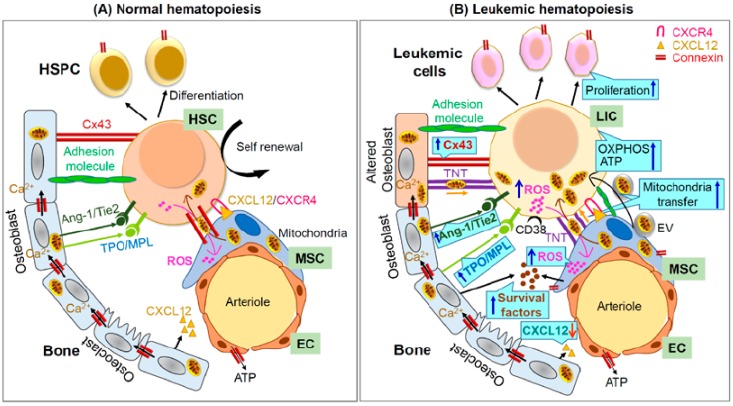
Schematic representation of normal and leukemic hematopoiesis. (**A**) Connexin (Cx) is expressed by hematopoietic stem and progenitor cells (HSPC) and bone marrow (BM) stromal cells. Gap junctions comprised of Cx43/45 between osteoblasts regulates CXCL12 secretion and HSC behavior within the niche. During myeloablation, Cx43 protects HSC from reactive oxygen species (ROS) induced damage by ROS scavenging through pseudosyncytial coupling of BM stromal cells. Cx43 mediated mitochondria transfer from BM stromal cells to HSPC regulates the emergency granulopoiesis and alleviates inflammation. Angiopoietin-1(Ang-1)/Tie2 and thrombopoietin (TPO)/MPL signaling maintains HSC quiescence. (**B**) Expression of Cx43 increased in leukemia initiating cells (LIC)/blast cells as well as in the surrounding BM niche cells. Leukemic cells receive signals from the BM microenvironment through diffusible paracrine factors (cytokines, chemokines, and metabolites) and/or by plasma-membrane fusion with tunneling nanotubes (TNT) or extracellular vesicles (EV). The higher expression of Cx43 in the leukemic environment by promoting the exchange of survival factors facilitates the proliferation and survival of malignant cells. High oxidative stress in leukemic cells further increases ROS levels in BM stromal cells and induces the transfer of mitochondria from stromal cells to LIC cells/blast cells through TNT and/or EV, while tumor cell CD38 promotes TNT formation. Transferred functional mitochondria regulate leukemic cells’ bioenergetics and metabolism, and are associated with leukemogenesis and chemoresistance. Although expression of connexins regulates TNT formation, their role in TNT assembly and organelle trafficking in the leukemic environment remains to be determined. BM stromal cells by modulating the TPO/MPL, Ang-1/Tie2, and CXCR4-CXCL12 pathways protect leukemic cells from chemotherapy. Blue arrow indicates increased expression in leukemia as compared to normal hematopoiesis. EC-endothelial cells. HSC:-Hematopoietic stem cells. MSC:-Mesenchymal stem cells, OXPHOS: Oxidative phosphorylation.

The GJ protein Cx43 is highly expressed in LT-HSC. Cx43 is dispensable for the steady-state hematopoiesis and its deficiency in HSC had no impact on steady-state HSPC differentiation and peripheral blood counts. Deficiency of p53 however results in the loss of HSC quiescence and a significant upregulation of Cx43 expression [77]. In contrast, expression of HSPC Cx43 is critical for hematopoietic reconstitution under stress hematopoiesis, and Cx43^-/+^ mice treated with cytotoxic drug, 5-FU (5-fluorouracil) show a delayed and ineffective hematopoietic response with an attenuated recovery of blood cells [14,15]. HSC are highly quiescent and preserve a low metabolic state to protect genomic integrity and retain functional capacity. Quiescent HSCs maintain low levels of reactive oxygen species (ROS), which supports their self-renewal and long-term repopulation ability. In contrast, a sustained and abnormal increase in ROS production during genotoxic stress and aging is associated with HSC differentiation and senescence. LT-HSC are more sensitive to oxidative stress than progenitors and differentiated cells [15,78,79]. Previously, our group has demonstrated that hematopoietic regeneration after 5-FU critically depends on HSPC Cx43 function (Figure 3A) [15]. Mechanistically, hematopoietic Cx43 regulates stress-induced hematopoietic regeneration following the transfer of 5-FU induced ROS from HSPC to the BMME, and prevents ROS-p38-p16/INK4a mediated HSPC senescence [15]. Interestingly, the deficiency of Cx43 in the BMME phenocopies the hematopoietic defect associated with Cx43 deficiency in the HSPC compartment. Cx43 GJ also mediate the communication between primary human CD34^+^ HSPC and BMSC [80], and engraftment of BM Cx43^+/-^ HSC is impaired in competitive transplantation models [14]. These findings suggest that BMME Cx43 expression is equally important for hematopoietic regeneration, and suggests that in stress hematopoiesis, heterocellular interactions between HSPC and BMME Cx43 are required for an adequate regenerative response. Expression of Cx43 is linked with constitutive inhibition of autophagy via direct interaction with autophagy related protein Atg16 and components of the phosphatidyl-inositide 3′-kinase (PI3K) autophagy initiation complex (Vps34, Beclin-1, and Vps15), while its elimination upregulates autophagosome biogenesis, even in nutrient-rich conditions [81]. In addition, functional Cx43 regulates mitochondrial integrity and metabolic activity in adipose tissue [82]. A large cohort of experimental data suggests that enhanced clearance of damaged mitochondria is a key mechanism in maintaining HSC stemness [83,84,85,86]. These findings raise the question of whether Cx43-deficiency mediated inhibition of hematopoietic regeneration depends on its effect on mitochondrial dynamics and fate, and warrant further investigation.

### 3.2. Gap Junctions in the HSC Niche

Stem cell niches are specialized microenvironments that critically regulate HSC maintenance and function. Considerable evidence has demonstrated the existence of functional GJIC in BMSC, osteoblasts, mesenchymal stem cells, and endothelial cells [16,17,54,87,88,89]. In addition, bidirectional traffic of cytosolic content through GJ was also observed between osteoblasts and BMSC, HSC and stromal cells, and between endothelial cells and lymphocytes/osteoprogenitors [71,80,90,91,92]. GJIC between stromal cells and HSC regulates the proliferation of HSC, while inhibition of transmembrane communication between HSC and stroma by amphotericin B reduces the ability of HSPC to form cobblestone-area forming cells and colony-forming units in culture [68]. Interestingly, Cx43 deficiency in BMSC results in a reduction of functional HSC and progenitor cells in the fetal liver, and impairs BM HSC proliferation and ST-HSC regeneration upon myeloablation, suggesting that stromal Cx43 acts as a critical regulator of hematopoiesis [16,17]. Analysis of transcript and protein expressions has demonstrated the presence of Cx30.3, Cx31, Cx31.1, Cx37, Cx40, Cx43, and Cx45 in different types of hematopoietic-supporting BM and fetal liver niche cells, with Cx43 being the most widely expressed and the major contributor to GJ communication in the BM microenvironment [17,93] (Table 1). Expression of Cx43 is 80-100 fold higher in neonatal BM as compared to normal adult mouse BM, and its expression upregulated in the endosteal space of the BM after 5-FU-treatment [18].

#### 3.2.1. Osteoblasts

Cells of the osteoblastic lineage are the predominant cells along the endosteum, and function as a critical regulator of HSC function in the BM [94,95]. In vitro experiments suggest that the osteoblast cells by modulating secretion of G-CSF and hepatocyte growth factor support the CD34^+^ human BM cells in long term culture [96,97]. Interestingly, osteoblasts co-transplanted with BMSC facilitate the engraftment of HSPC in an allogeneic environment [98]. Osteoblasts produce a wide array of molecules that are implicated in the maintenance of HSC, including (but not exclusively), osteopontin, which participates in HSC location and negatively regulates HSC expansion [99,100]; angiopoietin-1 (Ang-1), which regulates HSC quiescence through binding with Tie2 receptors [101]; and CXCL12 and vascular cell adhesion molecule 1 (VCAM-1), both of which are critical for the retention of HSPC within the BM [102,103] (Figure 3A). Enforced signaling through parathyroid hormone increases the number of osteoblastic cells and leads to Notch receptor ligand, Jagged 1, mediated increase in HSC numbers [94]. However, conditional ablation of osteoblast lineage after treatment with ganciclovir in Col2.3 Delta thymidine kinase transgenic mice (truncated form of the herpes virus thymidine kinase gene under the control of a 2.3 kb fragment of the rat α1 type I collagen promoter) shows profound alterations in hematopoiesis, including decreased number of HSC, lymphoid, myeloid, and erythroid progenitors in the BM, and progressive bone loss [104]. Cx43 is the most ubiquitous GJ protein in the BM mesenchymal lineage. It is expressed by chondrocytes, osteoblasts, osteocytes, and osteoclasts, and regulates osteoblastic differentiation and bone resorption and remodeling [105,106]. The loss of Cx43 in osteoblast lineage results in cortical thinning, increased marrow cavity area, and bone resorption due to abnormal osteoblast differentiation [107,108,109]. In a recent study, Lin et al. observed that the expression of Cx43 markedly increases during osteogenic differentiation [110]. BMSC Cx43 by upregulating the GSK-3β/β-catenin signaling pathways positively modulates the osteogenic differentiation, and plays a critical role in determining bone mass and bone mineral density [110]. Interestingly, Cx43, via ERK1/2 dependent recruitment of Sp1, regulates osteoprotegerin and restrains osteoclastogenesis and bone resorption [111]. The carboxy terminal domain of Cx43 serves as a docking platform for these signaling proteins (ERK1/2 and β-catenin) and is required for efficient bone remodeling. Of note, osteoblastic Cx43 is indispensable for the maintenance of the cellular content of the BM osteogenic microenvironment, and by modulating the content of CXCL12 in BM, it regulates the homing of HSC and progenitors in myeloablative recipients [112] (Figure 3A).

#### 3.2.2. Mesenchymal Stem Cells

Mesenchymal stem cells (MSC) constitute an essential HSC niche component and are able to self-renew and differentiate into bone, fat, and cartilage. BMSC wrap tightly around arterioles and more loosely around the sinusoidal blood vessels [95,113,114]. Expression profiling, lineage tracing, and in vivo passage analyses indicated that nestin^+^ perivascular cells were bona-fide MSC (nestin^+^ MSC), and were tightly associated with BM Lin^-^/CD48^-^/CD150^+^/CD41^-^ HSC and sympathetic nerve fibers in the close proximity of sinusoids [88,115]. Nestin^+^ MSC are a major contributor in the cellular niche involved in HSC maintenance, and they express and/or secrete cytokines and chemokines such as CXCL12, stem cell factor (SCF), IL-7, osteopontin, angiopoietin-1, and VCAM-1 [113,114,116,117]. The expression of both Cx43 and Cx45 is higher in nestin^+^ BM-MSC, and cooperatively regulates the secretion of CXCL12, which is essential for HSC retention in the BM, quiescence, and survival [8,88]. Schajnovitz et al. demonstrated the dual regulatory role of connexins in CXCL12 production, and suggested that (1) both Cx43 and Cx45 regulate the transcription of CXCL12 by nuclear Sp1 transcriptional activity, and (2) secretion of CXCL12 is cell contact-dependent and requires functional GJIC, while non-contacting BMSC can produce, but are unable to secrete CXCL12 because of a lack of intercellular communication [8]. In vivo inhibition of intercellular GJ formation by mimetic peptide without altering the expression of Cx43 and Cx45 attenuates the secretion of CXCL12 by blocking the calcium transmittance in neighboring BMSC, resulting in defective hematopoiesis and reduced homing of transplanted HSPC to the BM. Concomitantly, stress or G-CSF induced mobilization of HSPC is associated with the downregulation of Cx43 and Cx45 GJIC, which is accompanied by a lower secretion of CXCL12 in the BM [8] (Figure 3A).

#### 3.2.3. Endothelial Cells

BM endothelial cells (EC) line the interior of all blood vessels and regulate HSC expansion and the reconstitution of hematopoiesis after myeloablation [6,7,95,118]. EC expresses Cx37, Cx40, Cx43, and Cx45, which contribute to the regulation of blood flow, leukocyte adhesion and extravasation, vasomotor activity, angiogenesis, and the functional maintenance of vasculature [119,120]. The GJ protein, Cx43 is the major connexin expressed by EC and maintains the normal vascular function, and EC specific deletion of Cx43 in mice (EC-Cx43^Δ/Δ^ mice) results in hypotension, bradycardia, and compensatory hyperreninemia and hyperangiotensinemia [121]. Previous studies from our group have demonstrated the presence of an increased number of LT-HSC, ST-HSC, and progenitors in the peripheral blood of EC-Cx43^Δ/Δ^ mice secondary to hyperangiotensinemia, mostly angiotensin-II (Ang-II) [9]. Ang-II signals are mediated by two types of receptors, angiotensin type 1 receptor (AT1R) and type 2 receptor (AT2R). Ang-II, through both AT1R and AT2R in HSPC, and through AT2R in the EC, synchronously modulates cytoskeletal rearrangement and RhoA activity in HSPC and BM EC, which is associated with downregulation of active membrane β1-integrin expression, and results in HSPC de-adhesion and mobilization in the circulation [9]. Concomitantly, chronic Ang-II infusion increases HSPC and LT-HSC in the BM, augments myeloid differentiation, and attenuates the homing and engraftment potential of donor HSC [122]. 

BM sinusoid EC (SEC), defined as VEGFR3^+^/Sca1^-^/VE-cadherin^+^/VEGFR2^+^ constitute the cellular component extending through the largest surface area of the BM vascular niche, and play an essential role after chemo/radiotherapy by producing various factors such as Notch ligands, CXCL12, SCF, and pleiotrophin, that promote hematopoietic regeneration [6,123,124,125]. Of note, transplantation of SEC alone in irradiated mice rescues in vivo hematopoiesis and increases the frequency of surviving mice [126]. The regenerative potential of SEC is contact-dependent, and the angiocrine factors released by BM SEC through direct cell-to-cell contact with HSPC activate Notch signaling in HSPC and promote long-term ex vivo expansion of repopulating LT-HSC and in vivo reconstitution of the LT-HSC pool following myeloablation [6,7]. Similarly, human BM CD34^+^ progenitors exposed to irradiation and co-culture with EC show recovered reconstitution potential, while irradiated CD34^+^ progenitors cultured with cytokines alone are unable to reconstitute, which further highlights EC contact-mediated regulation of HSC regeneration [127]. Guo et al. have shown that the deletion of the Notch ligand, Jagged 2, using endothelial cell-specific Cre strain (VE-cadherin) is dispensable for steady-state hematopoiesis, while it promotes hematopoietic recovery in myelosuppressive conditions by activating Notch2/Hey1 signaling in HSPC [128]. In a recent study, Chen et al. demonstrated that Apelin-expressing (Apln^+^) EC, representing ≈0.003% of all BM cells, mainly express Cx43 and Cx45 GJ proteins, Notch ligands, and pleiotrophin, and are indispensable for physiological homeostasis and hematopoietic reconstitution after myeloablation [129]. Apln^+^ EC substantially increases after irradiation, and in response to VEGF-A provided by transplanted BM cells, in particular by HSPC and LSK cells, promotes vascular generation and BM transplantation [129]. Interestingly, the permeability of different vessels (arterioles and sinusoids) affects the localization of HSC in the BM. BM arteriolar EC are less permeable and maintain HSC in a ROS^low^ quiescent state, whereas, HSC in the vicinity of leaky SEC have increased ROS levels, leading to their activation and augmenting their differentiation and migration capacity [130].

#### 3.2.4. Bone Marrow Macrophages

Besides stromal niche cells, BM resident macrophages, defined as Gr-1^-^CD115^int^F4/80^+^CD169^+^, representing ≈2.6% of total BM cells also promote the retention of HSPC in the endosteal niche through interaction with osteolineage cells and nestin^+^ MSC [131,132]. These cells are anatomically juxtaposed with endosteal osteoblasts and likely to play an important role in bone mineralization [133]. Expression of Cx43 and Cx37 has been observed in macrophages stimulated with receptor activator of NFκB ligand (RANKL), lipopolysaccharide (LPS), and pro-inflammatory cytokines TNFα and IFNγ [134,135,136]. The GJ protein, Cx43 regulates RANKL dependent osteoclastogenesis and genes critical for bone formation, while blocking of Cx43 GJ channel during osteoclastogenesis results in decreased osteoclast counts in the BM [136,137]. Expression of Cx37, also noted in osteoblasts, osteocytes, and osteoclasts, albeit at lower levels than Cx43, is critically involved in the regulation of osteoclast differentiation and fusion. However, Cx37 deficiency in mice is associated with arrest of osteoclast maturation and sustained increased in bone mass [138]. In addition, Cx37 GJ present on macrophages/monocytes inhibits leukocyte adhesion to the endothelium through the release of ATP into the extracellular space, resulting in inhibition of atherosclerotic plaque formation [139]. Previously, our lab has demonstrated that autophagy regulator p62, through regulation of IKK/NFκB/CCL4 pathways, plays an important role in the maintenance of the macrophage-osteoblast niche, which is indispensable for the retention of short-term repopulating and myeloid progenitor cells in BM [79,140]. Furthermore, direct cell-to-cell interaction between osteoblasts and BM macrophages is necessary for the osteoblast differentiation and the expression of the chemokine CCL4, which modulates CXCL12 dependent HSPC traffic in the BM [140]. The BM CD169^+^ M1 macrophages, but not the M0 and M2 macrophages, induce stromal CXCL12 production by acting specifically on the nestin^+^ niche cells, and promotes HSPC retention in the BM [132]. The crosstalk between macrophages and nestin^+^ MSC is mediated by macrophage-derived soluble factor oncostatin M, which through the MEK-p38-STAT3 pathway stimulates the expression of CXCL12 by nestin^+^ MSC [141]. In vivo depletion of macrophages either by using macrophage Fas-induced apoptosis transgenic mice, or by the administration of clodronate-loaded liposomes, resulted in reduced CXCL12 production in the BM and consequent mobilization of HSPC to the peripheral blood circulation [131].

Cx43 also represents an alternative system of cell communication in the stem cell niche and allows the exchange of small molecules and organelles between HSC and the BMME. In a recent study, Mistry et al. demonstrated that in response to emergency granulopoiesis, ROS-induced oxidative stress opens the Cx43 GJ channel via PI3K-Akt activation and enables mitochondria transfer from BMSC to HSC [142]. Our unpublished findings further suggest that the Cx43 in HSC, through transfer of healthy mitochondria from HSC to stromal cells, maintains the energetic balance of BMSC, and positively regulates BM mesenchymal and hematopoietic regeneration in myeloablative recipients [143], however further investigations are required to identify the trigger signal(s) involved in the mitochondria trafficking and whether cells with the transferred mitochondria intrinsically reprogram myeloablative hematopoiesis, or if it depends on cues from the surrounding microenvironment. The presence of undocked Cx43 hemichannels in BMSC is also relevant and mediates the transfer of different metabolites within the extracellular medium [12]. The release of extracellular nucleotides like ATP through Cx43 hemichannels subsequently activates purinergic receptors, which upon activation increase HSC expansion and engraftment [32,144,145].

In the last decade, there has been a significant qualitative change in our understanding of the role of connexins and GJ in blood formation. From merely descriptive reports in the 1990s and 2000s, to a renewed interest in understanding how GJ control specific functions of HSC in relation with redox control and associated senescence, proliferation and survival. GJ directly (in hematopoietic cells) or indirectly (in the hematopoietic microenvironment) seem to participate in crucial activities and be exquisitely required for hematopoietic regeneration upon stress. Ongoing studies are being focused on understanding the cellular and molecular mechanisms that control the communication mediated by GJ between HSC and progenitors and their surrounding microenvironment.

## 4. Role of Gap Junctions in Leukemic Hematopoiesis

Leukemia usually arises from the malignant transformation of one or several HSC or progenitors. A subpopulation of leukemic cells, able to initiate and/or propagate leukemia and associated with leukemia resistance and/or relapse has been called leukemia-initiating cells (LIC). During leukemogenesis, outcompeting clones of leukemic cells with pathogenic mutations develop and result in clinical disease.

LIC usually represent a rare subpopulation within the oligoclonal expansion of leukemias and are characterized by sharing some stem cell properties, like self-renewal, increased proliferation, and survival capabilities, while usually failing to allow a normal pattern of differentiation, resulting in differentiation arrest and accumulation in lympho-hematopoietic tissues. In general, proliferating leukemic progenitors do express high levels of Cx43 [62] probably due to their high proliferation rate and perhaps related to the frequent presence of p53 loss-of-function mutations [77].

In recent years, significant interest has arisen for the level of expression and function of connexins in the leukemic BMME/niches. The BMSC including fibroblasts, osteoblasts, MSC, and endothelial cells play a vital role in the development and progression of hematological malignancies and contribute to chemotherapy resistance [146]. Increasing evidence demonstrates that LIC physically interact with their surrounding BMME, and that intercellular communication with BMSC has a direct impact on leukemic hematopoiesis, and regulates leukemic stem/progenitor cell survival, proliferation, differentiation, and self-renewal [24,32,147,148,149] (Figure 3B). Interestingly, a strong expansion of leukemic CD34^+^ cells and leukemic cobblestone-area formation was observed ex vivo after culture on BMSC [150,151,152], suggesting that in addition to communication via cytokines and extracellular matrix proteins, the expansion of leukemic cells is also influenced by cues provided through cell-to-cell contact with the BMME. Of note, disruption of GJ by carbenoxolone (CBX, a glycyrrhetinic acid derivative) in the co-culture of acute myelogenous leukemia (AML) cells with BMSC reduces the chemoresistance favored by the leukemic niche and enhances apoptosis [153]. Although CBX is not a GJ specific inhibitor, it has been proposed that by intercalating into the plasma membrane and binding to the GJ connexons, CBX induces a conformational change that results in closure of GJIC channels [136,154]. Weber and Tykocinski observed that the direct contact of AML cell lines HL-60 and PBL-985 with KM-102 stromal cells inhibits leukemic cell differentiation, and this effect was mediated by a functional GJ channel [155]. Similarly, GJIC between stroma and leukemic lymphoblasts inhibits leukemic cell proliferation by retaining its quiescence, thus favoring chemoresistance [147]. Of note, in vitro studies using OCI-AML3 and OCIM2 AML cell lines suggest that the higher expression of Cx43 in OCI-AML3 cells acts as a tumor promoter, which exerts its effect by promoting the exchange of growth factors; or by facilitating malignant cell proliferation and survival signal [156]. In contrast, others have reported that reduced Cx43 expression or deterioration of GJIC in BMSC is associated with leukemogenesis, while the upregulation of Cx43 GJIC in BMSC after chemotherapy or transfection with the Cx43 gene induces caspase 3 and 7 mediated apoptosis, and enhances the efficacy of therapies in hematologic malignancies [157,158,159,160,161]. In a minimal residual disease mouse model, the relapse of leukemia was delayed when mice were transplanted with human umbilical cord blood progenitors overexpressing Cx43 [160]. An antiproliferative effect of Cx43 was also observed in the U937 AML cell line expressing the AML1-ETO fusion protein, and it was mediated by the accumulation of p27^kip1^ protein [162]. Notably, all-trans retinoic acid (ATRA), a natural derivative of vitamin A, by upregulating Cx43 expression and enhancing GJIC in leukemic niche derived BMSC, arrests leukemic cell proliferation and induces apoptosis [157].

The transcript of several connexins including Cx26, Cx32, Cx37, Cx43, and Cx45 has been observed in primary human AML blasts, and the higher surface expression of Cx43 and Cx45 was found in most differentiated FAB M4 and M5 cells [163]. Higher expression of Cx45 associated with the altered regulation of the mitogen-activated protein kinase (MAPK) pathway and the release of pro-inflammatory cytokines IL-17, TNFα, and IFNγ, resulted in a pro-tumorigenic environment and protected AML cells from chemotherapy. Conversely, expression of Cx32 and Cx35 was comparable in both undifferentiated (FAB M0, M1, and M2) and differentiated (FAB M4 and M5) AML cells [163]. Furthermore, higher expression of Cx25 and Cx40 in acute lymphoblastic leukemia (ALL) and AML cell lines, as well as in AML patient’s cells play an important role in leukemia cell communication and chemoresistance. Inhibition of Cx25, however, decreases leukemic cell proliferation and sensitizes the cells to chemotherapy [153,161]. In a recent study, Kouzi et al. delineate a higher expression of Cx25, Cx31.9, and Cx59 in AML blasts and BM CD34^+^ cells, while the differential expression of these connexins was independent of the cytogenetic or molecular status of AML cells. Of note, a higher level of transcription of Cx25, Cx26, Cx30, Cx31, Cx32, Cx36, Cx37, Cx40, Cx46, and Cx62 was observed in AML BMSC [153]. Likewise, a higher expression of Cx43 was detected in multiple myeloma (MM) cell lines (RPMI8226, U266, and XG7), primary cell as well as in BMSC. The upregulation of Cx43 in BMSC plays a crucial role in the adhesion and migration of MM cells [164]. Functional Cx43 GJIC between MM cells and BMSC induces the release of IL-6, stromal cell derived factor (SDF)-1α, and IL-10, and associated with tumor cell proliferation and chemoresistance [165]. Inhibition of Cx43 GJIC by the non-specific GJIC inhibitors heptanol or 18α-glycyrrhetinic acid significantly attenuates CXCL12 secretion by BMSC, and augments bortezomib induced MM cell apoptosis [164,165] (Table 1). These findings, while not confirmed by genetic studies, may suggest a specialized GJ interaction (homo or heterotypic) between leukemia cells and BMSC in the leukemic microenvironment, and a potential role for GJIC in leukemic cell expansion and chemoresistance.

The BMME plays a critical role in the maintenance and retention of leukemic cells, and studies from our group have demonstrated that HSPC Cx43 GJ plays a protective role during stressful conditions and facilitates the transfer of potentially lethal ROS to the hematopoietic microenvironment following myeloablation, and prevents ROS mediated HSPC damage [15,79]. Interestingly, the ex vivo co-culture of AML cells with BMSC modifies cellular energy metabolism and increases the apoptotic threshold (chemoresistance) in leukemia cells, which was accompanied by the upregulation of anti-apoptotic protein, BCL2 and mitochondrial uncoupling protein, UCP2 [23,24]. Although the cancer cells largely depend on glycolytic metabolism to generate ATP, others emphasize the importance of oxidative phosphorylation in the tumor environment. The metabolism of LIC and AML blasts largely depends on mitochondrial oxidative phosphorylation to generate ATP for leukemic cell proliferation and survival [21]. Leukemia cells without mitochondrial DNA (ρ0 cells) show decreased tumor progression as compared to their parental counterparts. Co-culture of ρ0 cells with BMSC, however, shows increased tumor progression, which might be associated with the acquisition/transfer of mitochondrial DNA from the stromal cells [166]. Interestingly, CBX induced disruption of functional GJ in the leukemic niche results in decreased oxidative phosphorylation in AML cells, revealing a major perturbation in mitochondrial function, and in increased chemosensitivity and apoptosis of leukemic cells. Its pro-apoptotic effect was synergized with chemotherapy drug cytarabine (AraC) [153]. These findings suggest a link between efficient tumor formation and recovery of mitochondrial respiration, and GJ participation in the protective effect offered by the leukemic niche via trafficking of whole functional mitochondria, facilitating leukemogenesis. Since CBX is not a GJ specific inhibitor [154], further analyses of existing and to-be-generated genetic mouse knockout and knockin models modifying Cx expression are required to specify the role of Cx in leukemia progression and relapse.

### Mitochondria Trafficking Mediated by Connexins in Leukemia

Mitochondria are the key regulators of cellular bioenergetics and metabolism, and recent lines of evidence indicate that BMSC can rejuvenate damaged cells by mitochondrial transfer [166,167,168]. In the nonmalignant setting, BM-derived MSC protects against acute lung injury by restituting alveolar oxidative phosphorylation and ATP production through Cx43 dependent alveolar attachment and mitochondrial transfer [169]. Importantly, calcium-binding mitochondrial Rho-GTPase, Miro1, and Miro2, which coordinate microtubule and actin-based mitochondrial movement and/or Cx43 mediated tunneling nanotube (TNT) formation associated with the mitochondrial donation from MSC to epithelial cells, alveolar cell bioenergetic improvement and amelioration of the epithelial injury [170,171]. Furthermore, Cx43 regulates TNT formation in HeLa cells [172] and induced pluripotent (iP)SC derived mesenchymal progenitors [171]. In a recent study, Mahrouf et al. elegantly describe the bidirectional exchanges of mitochondria between damaged cells and MSC [173]. In particular, mitochondria released from the damaged cells were engulfed and degraded by MSC, leading to the induction of heme oxygenase-1 (HO-1), which by enhancing the MSC mitochondrial biogenesis donates more functional mitochondria to injured cells, and improves the effectiveness of MSC based therapies [173]. Interestingly, mitochondrial transfer from iPSC derived MSC to epithelial cells via TNT alleviated airway inflammation in a mouse model of asthma, and Cx43 plays an important role in the regulation of TNT formation and mitochondrial transfer [171].

Transplants of mitochondrial DNA deleted murine B16 melanoma and 4T1 breast carcinoma cells (ρ0 tumor cells) in WT C57BL/6 mice recover respiratory function and tumorigenicity after in vivo acquisition of mitochondrial DNA from host stromal cells [167]. The use of transgenic mice expressing the red fluorescent protein in their mitochondria further provides the functional evidence of intact mitochondria transfer from the host tissues to ρ0 tumor cells [168]. Seminal work in ρ0 A549 lung cancer cells, demonstrated recovery in aerobic respiration after the transfer of active mitochondria from BMSC [166]. It has been shown that leukemic cells have a higher mitochondrial level compared to non-malignant HSC, and the mitochondrial DNA was strongly amplified during the transformation from the chronic phase [174,175]. Remarkably, horizontal transfer of mitochondria between the BMME and leukemic cells requires cell-to-cell contact and proceeds through TNT and/or endocytosis (Figure 2B). These transferred mitochondria are fully functional and capable of boosting mitochondrial metabolism in leukemic cells and provide a survival advantage following chemotherapy [146,176,177,178].

AML progression is enabled by the transfer of functional mitochondria from BMSC to AML blasts through tumor-derived TNT. This process was stimulated by the superoxide generated from NADPH oxidase-2 (NOX2) on the AML blast, which in turn stimulates ROS generation in BMSC, and induces pro-tumoral mitochondrial transfer from the stroma [176]. Chemotherapy further increases the already high oxidative stress of the leukemic microenvironment, enhances the transfer of mitochondria from BMSC to leukemic cells, and promotes AML proliferation and relapse [146,176]. While the donation of functional mitochondria had no apparent adverse effect on the metabolic health of BMSC, AML blasts develop an increase in mitochondrial mass which is further enhanced by increased mitochondrial biogenesis in BMSC through peroxisome proliferator-activated receptor gamma coactivator 1-alpha (PGC-1α), which is essential for sustained mitochondrial transfer to AML cells [179]. Concomitantly, the depletion of TNT formation by cytochalasin B or inhibition of ROS by N-acetyl cysteine (NAC), glutathione, and diphenyleneiodonium (DPI) in leukemic cells shows a significant decrease in AML blasts [176]. These findings suggest that strategies targeting mitochondrial transfer from BMSC through NOX2 and PGC-1α inhibition may emerge as an intriguing approach in the context of minimal residual disease after chemotherapy treatment. Increased mitochondrial bioenergetics and ATP production in MM is also associated with mitochondria transfer from BMSC via tumor-derived TNT, and tumor cell CD38 supports the formation of TNT [178]. Notably, TNT signaling between B-cell precursor ALL (B-ALL) and surrounding BMSC cells induces the secretion of pro-survival cytokines including IP10/CXCL10, IL-8, and MCP-1/CCL2, and protects leukemic cells from chemotherapeutic drugs [177,180]. MSC isolated from ALL patient BM specimens adopt an activated, cancer-associated fibroblast-like phenotype with cytoskeletal and gene expression changes and a high-level of pro-inflammatory cytokine secretion [177]. The activity of these activated MSC may be at the root of leukemia relapse by promoting leukemogenesis by transfer of mitochondria through TNT and prevent the rise of intracellular levels of ROS associated with chemotherapy with AraC and daunorubicin (DNR). Interestingly, reduction of mitochondria transfer by a microtubule-damaging drug, vincristine, or prevention of cancer-associated fibroblast activation by corticosteroids (a potent anti-inflammatory agent used for ALL therapy) selectively diminished the leukemia burden and improved survival, these were synergistic with AraC and DNR [177]. Of note, in a disseminated mouse model of ALL, AraC treatment induces activation of MSC, increases mitochondrial mass and mitotransfer, wherein the association of human CD19^+^ cells with a nestin^+^ BMSC was observed [177]. A higher number of nestin^+^ MSC was also found in AML patient’s BM, and they represent cell-to-cell contact-dependent ROS detoxifying mechanism and mitochondria transfer, to allow the chemoresistance in AML cells. Combining nestin^+^ MSC depletion and chemotherapy, however, exaggerated the elimination of BM leukemic cells, and could be used as an effective approach to eradicate the residual disease after chemotherapy treatment [181].

## 5. Conclusions and Future Perspectives

The contribution of the BMME has gained increasing attention and the role of mitochondria transfer in leukemia progression and chemoresistance is being highlighted. However, the differential role of the BMME cellular component in mitochondrial transfer, and the fate of transferred mitochondria in leukemic cells remains unclear. The emergence of new pharmacological and molecular approaches targeting the depletion of intercellular organelle exchange could be of particular clinical interest in leukemia treatment, and they could synergize the effect of chemotherapy drugs in eradicating minimal residual disease after chemotherapy. However, the key challenge is the dual role of the BMME in regulating normal and malignant hematopoiesis, since inhibiting the leukemic cell’s development must be followed by the re-establishment of normal hematopoiesis. Connexin plays a significant role in leukemia pathophysiology, and the niche-induced chemoresistance depends on cell-to-cell contact and functional GJ communication. Cx43 mediated mitochondria transfer exerts a protective effect in a nonmalignant setting but the mechanisms controlling this effect are not understood. Further studies are required to evaluate the connexin dependent regulatory mechanism involved in leukemia relapses and identify whether the protective effect of connexins associated with mitochondria transfer derives from specific leukemic microenvironment(s) and can be inhibited to prevent leukemia growth. On the other hand, enhanced connexin activity may result in increased hematopoietic engraftment in the setting of stem cell transplantation or post-chemotherapy recovery, suggesting that the activation and/or inhibition of connexin activity may provide complementary roles in the therapy of patients with hematological malignancies. Since different connexin isoforms have diverse GJ channel-dependent and -independent functions, and their expressions are modified in different stages of leukemia development, the focus for new strategies should be to identify stage-specific regulation of connexins during leukemic clonal evolution and their involvement in leukemia physiology.

## Figures and Tables

**Table 1:** Expression of different connexins and their function in normal and leukemic hematopoiesis.

Cell Type	Connexin Type	Function	Ref.
**Normal HSC and BM Stromal Cells**			
LT-HSC and progenitors	Cx43, Cx45, Cx31, Cx31.1, Cx32, Cx37, and Cx50	Expression	[15,28]
LSK and LK cells	Cx32	Maintain HSPC quiescence and stemness	[29]
HSPC	Cx43	Reduces HSC senescence via ROS transfer to BMSC during stress induced hematopoietic regeneration	[14,15]
BM stromal cells (Osteoblasts, MSC and endothelial cells)	Cx31	Expression	[93]
BM stromal cells (Osteoblasts, MSC and endothelial cells)	Cx43 and Cx45	Regulates CXCL12 secretion, HSC growth, differentiation and homing	[8,17,112,142]
BM stromal cells	Cx43	1) Determine bone mass and bone mineral density by modulating osteogenesis	[16,110,142,182]
		2) Mitochondria transfer from BMSC to HSC and emergency granulopoiesis	
		3) HSPC proliferation and differentiation of myeloid blood cell precursor	
		4) Hematopoietic regeneration after chemotherapy	
BM Endothelial cells	Cx43	Normal vascular function, leukocyte adhesion and transmigration	[9,183]
**Leukemia Cell Lines, Primary Cells, and Leukemic BM Stromal Cells**			
OCIM2 and OCI-AML3 cells	Cx43 and Cx32	↑Cell proliferation	[156]
CCRF-CEM lymphoblast cells	Cx33, Cx40, Cx43, Cx45, Cx46, and Cx50	↑ Chemoresistance	[147]
		↓Apoptosis	
		↓Differentiation	
HL-60 and PBL-985 cells	GJ	↓Differentiation	[155]
U937 AML cells expressing AML1-ETO fusion protein	Cx43	↓Cell proliferation	[162]
KG-1, KG-1a, HL-60, OCI-AML3, MV4-11, MoLM-13 Jurkat, and THP1 cells	Cx25, Cx31.9, Cx40, Cx43, Cx45, and Cx59	↑Cell proliferation	[161]
		↑Chemoresistance	
Primary AML cells	Cx26, Cx32, Cx37, Cx43, and Cx45	↑ Chemoresistance	[163]
		↓Apoptosis	
AML-blasts and BM CD34^+^ cells	Cx43, Cx45, Cx25, Cx31.9, and Cx59	↑Cell proliferation	[153,163]
		↑Chemoresistance	
AML BM-stromal cells	Cx25, Cx26, Cx30, Cx31, Cx32, Cx36, Cx37, Cx40, Cx46, and Cx62	↑Cell proliferation	[153]
		↑Chemoresistance	
Primary Multiple Myeloma (MM) cells and cell lines (RPMI 8226, U266, and XG-7)	Cx43	Adhesion and migration of MM cells	[164,165]
		↑Cell proliferation	
		↑Chemoresistance	
Multiple Myeloma BM-stromal cells	Cx43	Adhesion and migration of MM cells	[164,165]
		↑Cell proliferation	
		↑Chemoresistance	
Cx32-KO mice		↑Leukemia incidence	[76]

## Abbreviations

Atg16	Autophagy related protein 16
Akt	Protein kinase B
CCL4	Chemokine C-C motif ligand 4
CXCL12	C-X-C motif chemokine 12
CXCR	C-X-C chemokine receptor
ERK	Extracellular Signal-regulated Kinase
FAB	French-American-British classification
G-CSF	Granulocyte colony stimulating factor
GSK-3β	Gycogene Synthase Kinase-3-beta
IFNγ	Interferon gamma
IKK	Inhibitory kappa B kinase
IL-7	Interleukin 7
iPSC	Induced Pluripotent Stem Cells
LSK	Lin-/cKit+/Sca1+
MEK	Mitogen-activated protein kinase kinase 1
NADPH	Nicotine adenine dinucleotide phosphate, reduced form
NFκB	Nuclear factor kappa B
PDZ	domain shared by the postsynaptic density protein (PSD95), Drosophila disc large tumor suppressor (DlgA), and zonula occludens-1 protein (ZO-1)
Sp1	Specificity protein 1
STAT3	Signal Transducer And Activator Of Transcription 3
TNFα	Tumor Necrosis Factor alpha
TPO/MPL	Thrombopoietin/Myeloproliferative Leukemia protein (TPO receptor)
VEGFR	Vascular Endothelial Growth Factor Receptor
Vps34	Vacuolar Protein Sorting 34
